# IκK-16 decreases miRNA-155 expression and attenuates the human monocyte inflammatory response

**DOI:** 10.1371/journal.pone.0183987

**Published:** 2017-09-14

**Authors:** Norman James Galbraith, James Burton, Mathew Brady Ekman, Joseph Kenney, Samuel Patterson Walker, Stephen Manek, Campbell Bishop, Jane Victoria Carter, Sarah Appel Gardner, Hiram C. Polk

**Affiliations:** The Price Institute of Surgical Research, The Hiram C. Polk, Jr., M.D. Department of Surgery, University of Louisville School of Medicine, Louisville, KY, United States of America; National Institutes of Health, UNITED STATES

## Abstract

Excessive inflammatory responses in the surgical patient may result in cellular hypo-responsiveness, which is associated with an increased risk of secondary infection and death. microRNAs (miRNAs), such as miR-155, are powerful regulators of inflammatory signalling pathways including nuclear factor κB (NFκB). Our objective was to determine the effect of IκK-16, a selective blocker of inhibitor of kappa-B kinase (IκK), on miRNA expression and the monocyte inflammatory response. In a model of endotoxin tolerance using primary human monocytes, impaired monocytes had decreased p65 expression with suppressed TNF-α and IL-10 production (P < 0.05). miR-155 and miR-138 levels were significantly upregulated at 17 h in the impaired monocyte (P < 0.05). Notably, IκK-16 decreased miR-155 expression with a corresponding dose-dependent decrease in TNF-α and IL-10 production (P < 0.05), and impaired monocyte function was associated with increased miR-155 and miR-138 expression. In the context of IκK-16 inhibition, miR-155 mimics increased TNF-α production, while miR-155 antagomirs decreased both TNF-α and IL-10 production. These data demonstrate that IκK-16 treatment attenuates the monocyte inflammatory response, which may occur through a miR-155-mediated mechanism, and that IκK-16 is a promising approach to limit the magnitude of an excessive innate inflammatory response to LPS.

## Introduction

A frequent complication in patients following trauma, major surgery, or sepsis is secondary infection [1]. Impaired pathogen clearance may occur due to defective host defense mechanisms as a result of such a significant physiological insult to the body [2]. Genomic, transcriptomic, and proteomic studies in trauma patients show that the magnitude of the immunologic response after an insult, such as trauma, correlates with a higher risk of subsequent complications [3, 4]. Impaired monocyte function is one facet of immune dysregulation that continues to be recognized as a central tenant of trauma- or sepsis-related immunosuppression [5, 6]. However, efforts to pharmacologically modulate this response have failed to consistently improve mortality and have, therefore, not secured a place in the armory of the compleat surgeon [7, 8].

IκK-16 is a new therapy recently studied in animal models of sepsis and shock [9, 10] that reduces nuclear factor-κB (NFκB) activation by selectively blocking inhibitor of kappa-B kinase (IκK), and thus decrease the magnitude of the pro-inflammatory response. The beneficial effects of IκK-16 have included decreased lung injury, renal dysfunction, and cardiac failure; however, this treatment has not been applied to humans [11, 12]. A better understanding of how IκK-16 affects intracellular mechanisms in human leukocytes is required.

microRNAs (miRNAs) are increasingly recognized as important regulators of immune function. These small non-coding RNAs are stimulated and released into the intracellular and extracellular environment by various triggers such as pathogen- and danger-associated molecular patterns (DAMPs and PAMPs). The stability of miRNAs has placed miR-based biomarker research at the forefront of contemporary cancer and sepsis-related academic inquiries [13, 14]. One of the most commonly studied miRNAs is miR-155, which is expressed in myeloid cells and is upregulated in response to lipopolysaccharide (LPS) stimulation to toll-like receptor (TLR)-4 receptors [15]. miR-155 inhibits the translation of suppressor of cytokine signaling-1 (SOCS1) and Src-homology region 2 domain-containing phosphatase-1 (SHP-1) mRNA, among other target genes, into functional proteins. As a result, this post-transcriptional modification releases the suppression on signaling pathways, resulting in the promotion of the pro-inflammatory response [16, 17].

Our group has previously demonstrated the important role and regulation of miR-155 in immune dysfunction during hypothermia using a model based on primary human monocytes. In the current study, we have adopted a model of impaired monocyte function, or endotoxin tolerance, which is representative of the cellular hypo-responsiveness observed in some surgical patients [18, 19]. Our initial data demonstrated that miRNA-155 was consistently upregulated in the impaired monocyte. We hypothesized that IκK-16 would decrease the expression of miR-155 and decrease monocyte cytokine production in response to LPS.

## Materials and methods

### Subjects and monocyte isolation

This study was approved by the University of Louisville Institutional Review Board. Blood from healthy volunteers was collected in EDTA vacutainers (Becton Dickinson, Franklin Lakes, NJ). Anti-inflammatory medication, or the presence of acute illness or chronic disease, was cause for exclusion.

Human Whole Blood CD14 Microbeads (Miltenyi Biotec, Auburn, CA) were used for positive selection of monocytes according to the manufacturer’s instructions. Monocytes were >95% purified as determined by flow cytometry and were manually counted with Trypan Blue staining to ensure that cells were >95% viable. Monocytes were cultured in RPMI 1640 (Sigma Aldrich, St Louis, MO) and supplemented with 10% fetal bovine serum, L-glutamine, and antibiotic/antimycotic agents (Thermo Fisher Scientific, Waltham, MA) in an incubator with 5% CO2 at 37°C. All cultures occurred in cell suspension using 50 mL polypropylene tubes containing 1 x 10^6^ cells per condition. In the model of endotoxin tolerance, following isolation and 1 h of rest, monocytes were treated for 16 h with either media only (naïve control) or 10 ng/mL of LPS (impaired) (Escherichia coli 0111:B4; Sigma Aldrich, St. Louis, MO). At 17 h following isolation, monocytes were centrifuged, washed, counted, and re-suspended in fresh media at a concentration of 0.5 x 10^6^ per mL. Cells were then stimulated with a 100 ng/mL LPS challenge to determine the inflammatory response. At the indicated time points, supernatant was collected and stored at -80°C until further analysis. For experiments using IκK-16, cells were treated with either IκK-16 or DMSO (control) for 1 h after isolation before stimulation with 100 ng/mL of LPS for an additional 16 h before analysis.

### RNA isolation

At the indicated time points, cells were pelleted and stored in 300 μL of lysis buffer at -80°C. RNA was extracted using the MirVana miRNA isolation kit (Thermo Fisher Scientific, Waltham, MA). For quality control testing, RNA purity and concentration were measured using a Nanodrop N-1000 spectrophotometer (Agilent Biosystems, Santa Clara, CA). Samples were used only when they fulfilled the quality control criteria of 1.8–2.2 purity, based on the ratio of absorbance at 260 nm and 280 nm (A260/A280 ratio).

### Messenger RNA analysis

Complementary DNA (cDNA) was synthesized by reverse transcription (High Capacity cDNA Reverse Transcription kit, Life Technologies, Foster City, CA). For TNF-α, IL-10, SOCS1, and SHP1 mRNA quantification, specific primers were used for qRT-PCR, with data normalized to 18S. For TLR mRNA expression profiling, Taqman® Array Human Toll Pathway Core Components profiling plates (Life Technologies, Foster City, CA) were used with a combination of 18S, GAPDH, and beta2-microglobulin for normalization. A ΔRn (normalized reporter value) threshold of 0.1 was selected. Taqman® Universal Master Mix was used with the StepOne Plus RealTime-PCR-System instrument for qRT-PCR (Applied Biosystems®, Foster City, CA). Fold changes were calculated using the ΔΔCT method, comparing the impaired monocyte relative to the naïve monocyte as its control [20].

### MicroRNA analysis

For microRNA screening, a total of 1500 ng of diluted RNA was incorporated to create cDNA by reverse transcription with Megaplex Reverse Transcription Human Pool A v2.1 (Life Technologies, Foster City, CA). Subsequently, the cDNA was loaded into Taqman® Low Density Array cards, divided between 384 wells, for qRT-PCR (human card A, Life Technologies, Foster City, CA) on a ViiA™ 7 Real-Time PCR System (Applied Biosystems®, Foster City, CA). RNU6B, RNU44, and RNU48 were used in combination as endogenous controls for data normalization. For single assay microRNA quantification, specific primers were used per microRNA for reverse transcription to create cDNA, with specific primers used for qRT-PCR with Taqman® Universal Master Mix. RNU6B was used as an endogenous control for data normalization, and all assays were run in duplicate on a StepOne Plus RealTime-PCR-System instrument (Applied Biosystems®, Foster City, CA). All microRNA data was analyzed using a cycle threshold of 0.1 by the ΔΔCT method.[20]

### Western blot analysis

Cells were pelleted and lysed using RIPA buffer supplemented with protease and phosphatase inhibitors (Thermo Scientific, Rockford, IL) and stored at -80°C until subsequent analysis. Protein samples were sonicated using a Sonifier 250 (Branson Ultrasonics, Danbury, CT) and collected by centrifugation at 10,000g for 10 mins. Total protein concentrations were quantified using a bicinchoninic acid (BCA) protein quantification assay (Thermo Scientific, Rockford, IL). Protein samples of 30 μg were loaded into gradient 4–12% Bis-Tris Plus 12-well gels in combination with 4X Bolt^TM^ LDS sample buffer with 1:100 2-mercaptoethanol. Gels were run in a Bolt^TM^ Mini Gel Tank in Bolt MES SDS running buffer (all from Thermo Scientific, Waltham, MA) at 180 mV for 45 min, which included a protein size standard (Protein Western C Standard, Bio Rad, Hercules, CA). Proteins were transferred to a nitrocellulose membrane using iBlot Gel Transfer Stacks and an iBlot transfer device (Fisher Scientific, Hampton, NH). Membranes were blocked with 5% non-fat dried milk in TBS-T for 1 h at room temperature and incubated overnight with primary antibodies (either rabbit, mouse or goat anti-human as appropriate) at room temperature. All antibodies were purchased from Cell Signaling (Danvers, MA), except SOCS1 where antibodies from Abcam were used (Cambridge, UK). Primary antibody concentrations were 1:10,000 for beta-actin and 1:1000 for p65, vinculin, SOCS1 and SHP1. Membranes were washed in TBS-T before incubation with HRP-conjugated secondary antibodies (goat anti-rabbit, horse anti-mouse or Donkey anti-goat as appropriate) at a concentration of 1:1000 for 1 h at room temperature. After further washing, protein bands were demonstrated with Clarity Western ECL Substrate (Bio-Rad, Hercules, California), and membranes were imaged using a ChemiDoc MP (Bio Rad, Hercules, CA). Protein densitometries were measured using ImageLab software (Bio Rad, Hercules, CA). For quantification, p65 was expressed as relative density units as a ratio over beta-actin as the loading control.

### Cytokine analysis

Tumor necrosis factor-alpha (TNF-α) and interleukin (IL)-10 were analyzed in duplicate using enzyme-linked immunosorbent assays, according to the manufacturer’s instructions (eBioscience, San Diego, CA). A standard curve from recombinant human cytokines was used to calculate supernatant cytokine protein concentrations. Sensitivities for the lower limits of detection were concentrations of 4 pg/mL and 2 pg/mL for TNF-α and IL-10, respectively.

### IκK inhibition

IκK-16 was purchased from Sigma Aldrich, St. Louis (MO). Compounds were reconstituted from stock solution in DMSO as per manufacturer’s instructions and diluted in media to the appropriate concentrations, resulting in a final DMSO concentration of 0.01%. For the non-IκK-16 treated control, the equivalent DMSO concentration was used. A dose of 100 nM of IκK-16 was used unless otherwise specified.

### Transfection experiments

Primary human monocytes were transfected using the N-TER Nanoparticle siRNA Transfection System (Sigma-Aldrich) according to the manufacturer's instructions. After initial treatment of control (DMSO) or 100 nM IκK-16 for 1 h, monocytes were transfected with miR-155 mimics or antagomirs (Life Technologies, Foster City, CA, USA) at a concentration of 40 nM/well for 16 h. Control cells were transfected with scramble miRs as a negative control (sham). After 16 h of transfection, the medium was replaced with fresh medium and the cells were stimulated with 100 ng/mL LPS for an additional 6 and 24 h. Cells were collected for RNA isolation, whereas supernatants were collected for cytokine measurements. Effective transfection was confirmed by determining miR-155 levels in isolated RNA by qRT-PCR, using RNU6B as described, ensuring upregulation of miR-155 cells transfected with mimics and downregulation of miR-155 in cells transfected with antagomirs, relative to sham conditions.

### Statistical analysis

Data are shown as mean ± S.E.M. Each donor was used as its own control. Normally distributed data were analyzed using paired T-tests, and data that were not normally distributed were analyzed by the Wilcoxon-signed rank test. Data with multiple comparisons were analyzed by repeated measures analysis of variance with Tukey’s test for post hoc analysis. Significance was set at P < 0.050, with the exception of miRNA screening data in which significance was set at P < 0.010. SigmaPlot was used for presentation of data and for statistical analysis (SyStat Software, Inc., Chicago, IL).

## Results

### Low dose LPS stimulates an initial pro-inflammatory response but with subsequent NFκB and cytokine suppression

**Fig 1:**
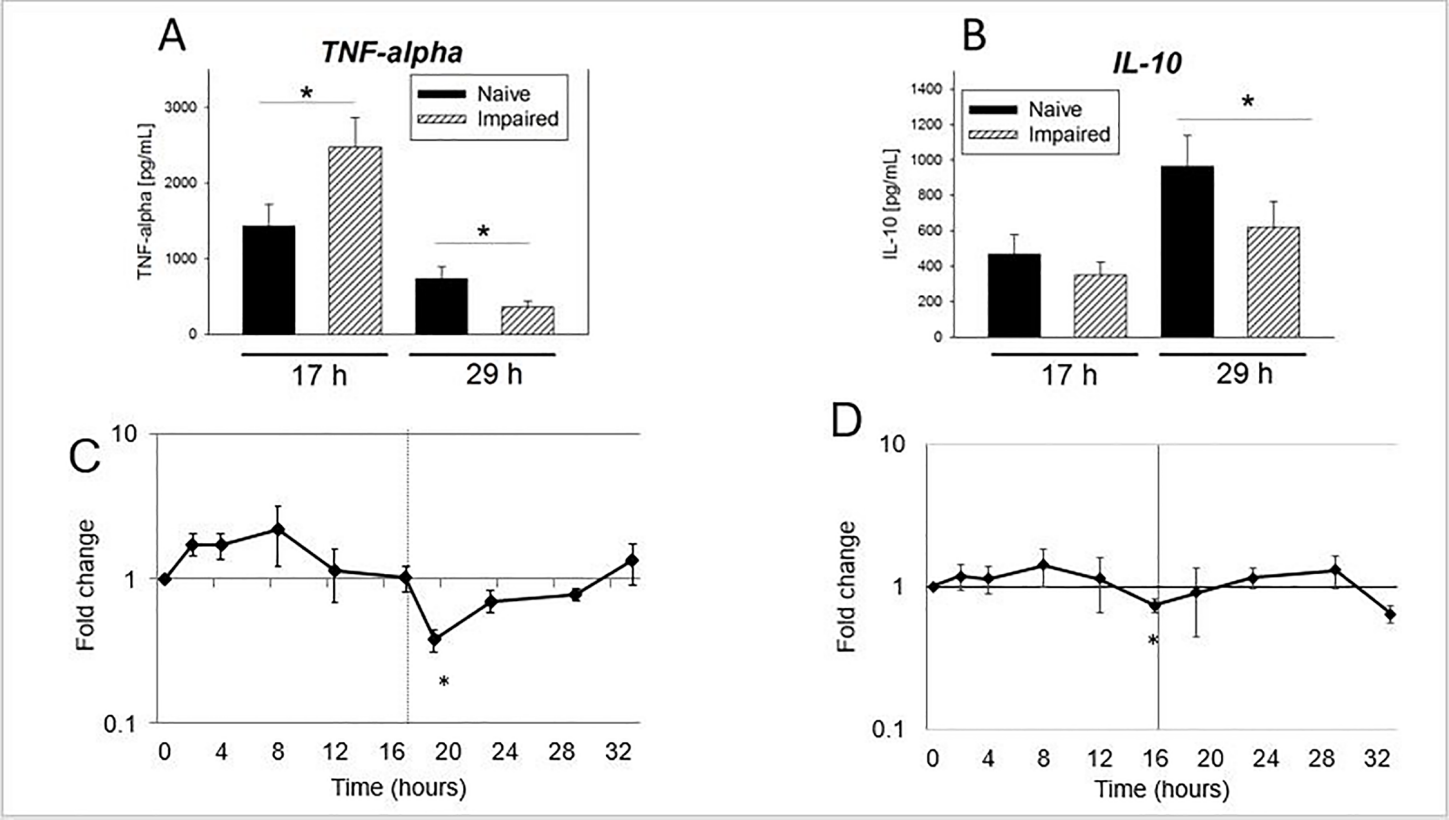
(A) Supernatant TNF-α concentrations and (B) IL-10 concentrations comparing naïve (no LPS pre-treatment) and impaired (10 ng/mL LPS pre-treatment) conditions. Time points are shown for the end of the pre-treatment prior to the LPS challenge (17 h), and then the response 12 h after the LPS 100 ng/mL challenge (29 h). Levels were determined by ELISA. N = 8. Mean ± S.E.M. are shown. *P < 0.05, paired T-test. Time course of TNF-α (C) and IL-10 (D) gene expression in the impaired monocyte. Monocytes were treated with 10 ng/mL of LPS given after 1h for a duration of 16 h. At 17 h, cells were washed and resuspended in fresh media, then received 100 ng/mL of LPS at 17 h. Levels were measured by qRT-PCR, and expressed as fold change (2^-ΔΔCT^) relative to the naïve monocyte. Mean ± S.E.M. are shown. N = 4. *P < 0.05. # P = 0.09, paired T-test.

A model of endotoxin tolerance was developed to represent the suppressed immune response. Monocytes that were treated with 10 ng/mL of LPS demonstrated an increased level of TNF-α production at 17 h (P < 0.05) (Fig 1A). However, in response to a 100 ng/mL LPS challenge, the subsequent ability to produce TNF-α was markedly suppressed at 29 h (P < 0.05). Impaired conditions also demonstrated suppressed IL-10 production in response to the LPS challenge at 29 h (P = 0.04) (Fig 1B). Cell viability was equivalent between naïve and impaired conditions (S1 Fig).

**Fig 2:**
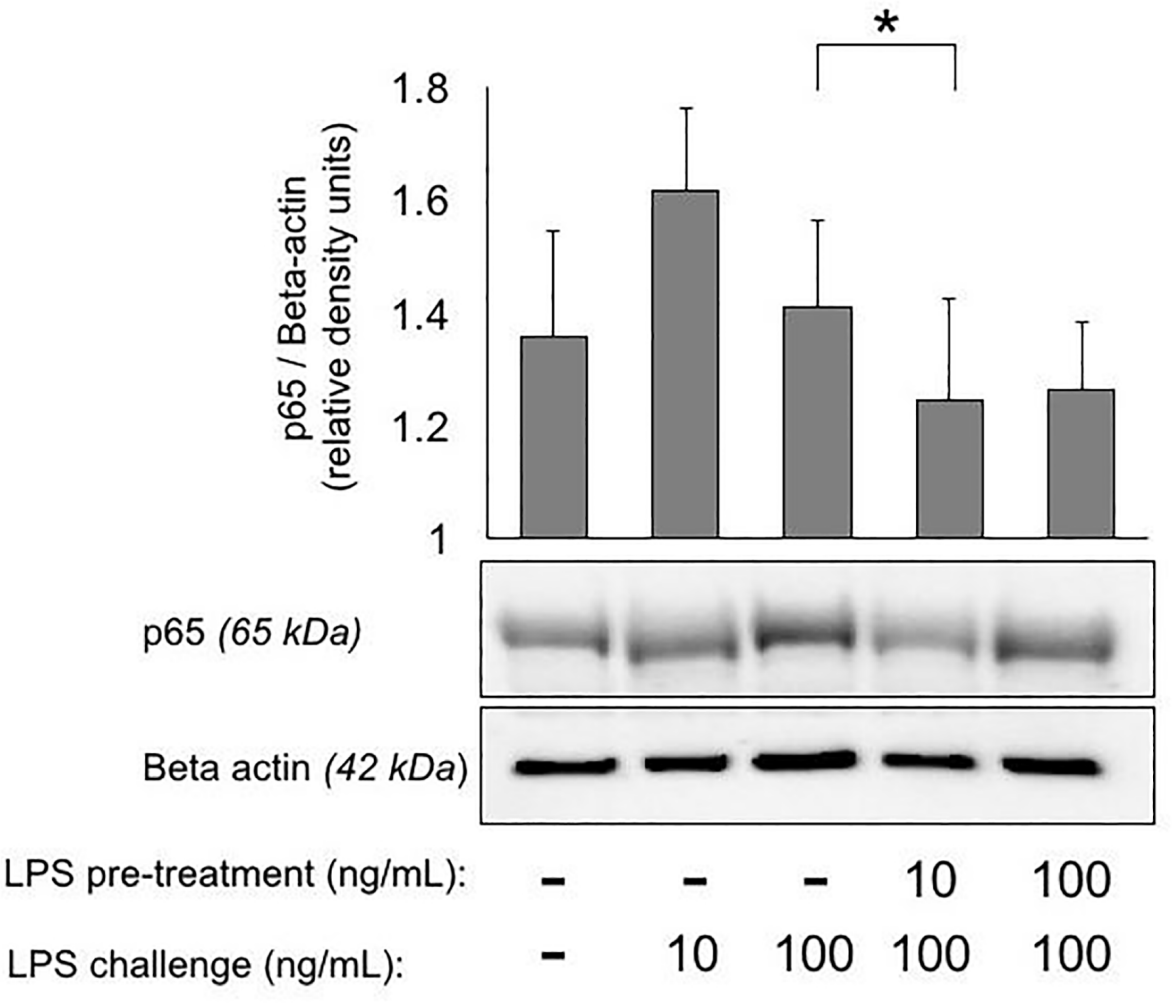
Expression of NFκB (p65) as measured by Western blot analysis. Cells given pre-treatment at 1 h after isolation until 17 h, and then were washed, suspended in fresh media, and given a further treatment (challenge) for 60 min. Quantitative data are expressed as relative density units, using beta-actin as a loading control. Naïve conditions (media only / 100 ng/mL LPS) and impaired (10 ng/mL LPS / 100 ng/mL LPS) were compared by paired T-test. N = 6. Mean ± S.E.M. *P < 0.05.

Cytokine gene expression and NFκB analysis of the impaired monocyte was undertaken to determine whether suppressed immune responses were due to alterations in canonical signaling in this model of endotoxin tolerance. A time course experiment to examine the gene expression of TNF-α in the impaired monocyte demonstrated an initial upregulation of TNF-α mRNA in response to 10 ng/L of LPS, observed maximally at 2 h (P = 0.09) (Fig 1C). However, after the 100 ng/mL LPS challenge at 17 h, a clear suppression of TNF-α mRNA levels was be observed maximally at 19 h compared to the naïve control (P < 0.05). Decreased IL-10 gene expression was observed in impaired cells at 17 h (Fig 1D, P < 0.05). Western blot analysis showed that impaired monocytes (10 ng/mL LPS/100 ng/mL LPS) had a decreased concentration of p65 (NFκB) at 60 min after the LPS challenge compared with naïve monocytes (media only/100 ng/mL LPS) (P < 0.05, Fig 2).

**Fig 3:**
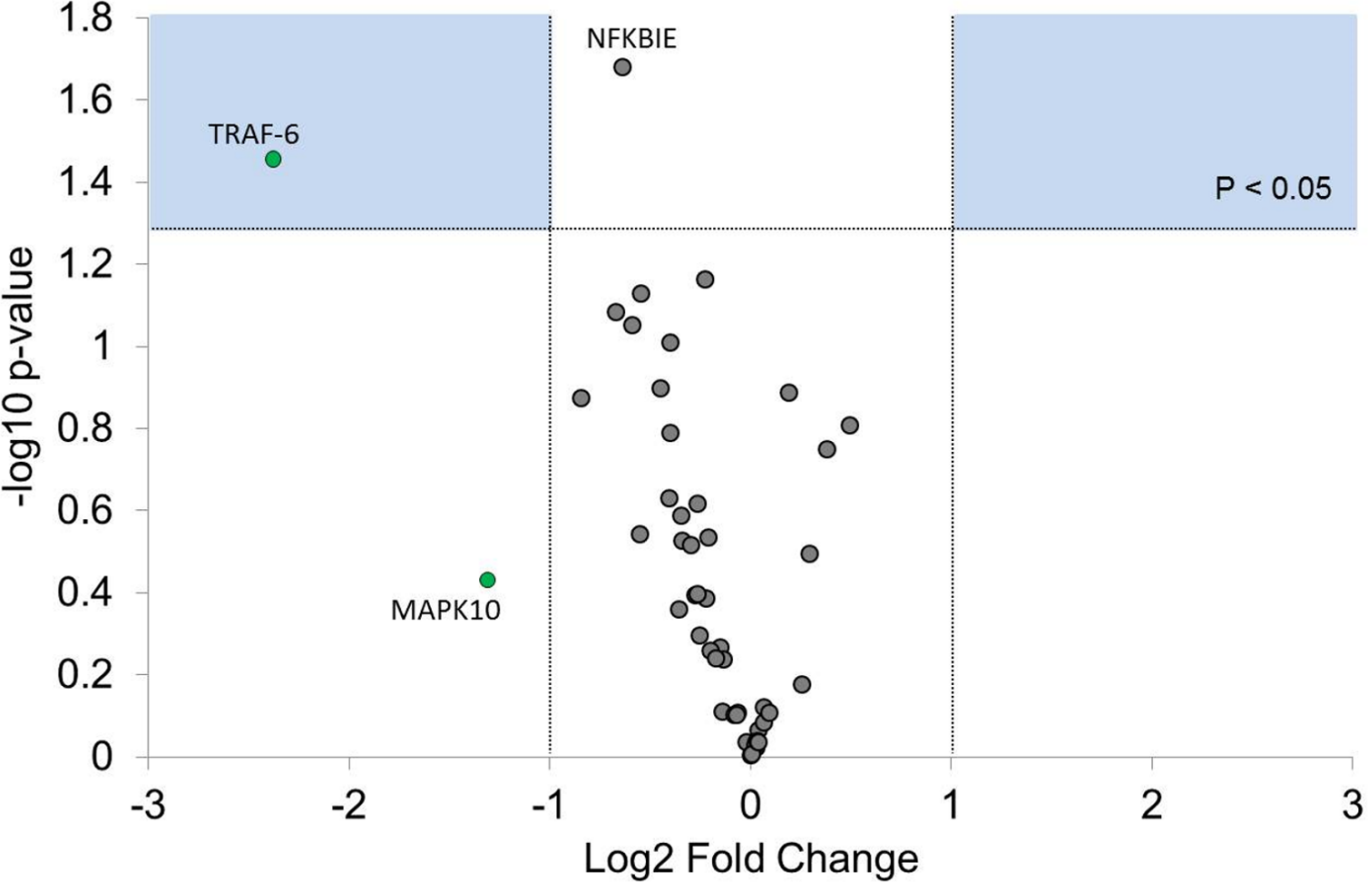
Volcano plot showing toll-like receptor (TLR) gene expression profiles, expressed the impaired monocyte relative to the naïve monocyte. Monocytes were cultured in media only (naïve) or 10 ng/mL LPS (impaired) until 17 h and then stimulated with an LPS 100 ng/mL challenge for 2 h. RNA was extracted from cell pellets, and 44 TLR genes were measured by qRT-PCR. Fold changes (FC) calculated by ΔΔCT method are expressed on the x-axis, with significance (-log10 p-value) demonstrated on the y-axis. Data were normalized to housekeeper genes 18S, GAPDH, and B2M. Green dots represent genes that are downregulated by a fold change of 0.66 (equivalent to fold regulation of -1.5). Dots in the blue boxes represent genes that were significantly dysregulated compared with the naïve monocyte (P < 0.05), paired T-test. N = 4. GAPDH = glyceraldehyde 3-phosphate dehydrogenase. B2M = beta-2 macroglobulin.

In order to characterize the transcriptional change in the impaired monocyte, alterations in upstream signaling were studied by profiling TLR gene expression (Fig 3). TNF receptor-associated factor 6 (TRAF-6) was significantly downregulated (P < 0.05). Other suppressed genes that did not reach statistical significance included NFKBIE, CD14, IKBKE, MAPK10, and REL.

### miR-155 is upregulated in the impaired monocyte

**Fig 4:**
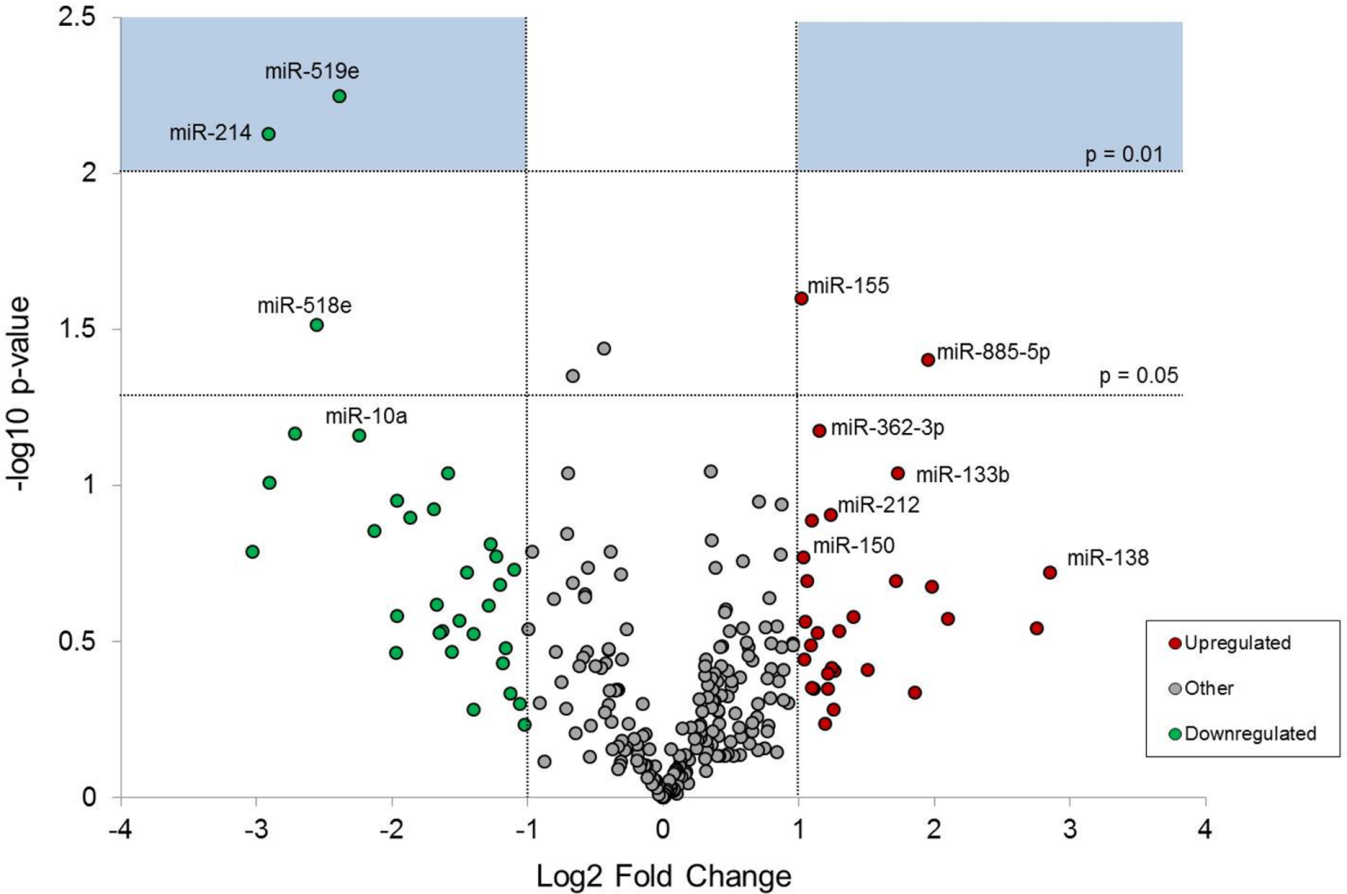
Volcano plot of miRNA screening of microRNA expression in the impaired monocyte relative to naïve conditions. Monocytes were cultured in media only (naïve) or 10 ng/mL of LPS until 17 h. RNA was collected at 17 h, immediately prior to a 100 ng/mL LPS challenge. Data were normalized to housekeeping genes RNU6B, RNU44, and RNU48. Green dots represent miRNAs that were downregulated by a log2 fold change of -1.0. Red dots represent miRNA’s that were upregulated by a log2 fold change of +1.0. miRNAs in the blue boxes represent those that reached statistical significance (P < 0.01), paired T-test. N = 5.

Differences in miRNA expression between naïve and impaired monocytes were initially determined by miRNA screening. Of the 360 miRNAs measured, those that reached statistical significance included miR-214 and miR-519e (P < 0.01) (Fig 4). Other miRNAs approaching significance with log2 fold changes up- or downregulation by greater than 1 included miR-10a, miR-133b, miR-138, miR-150, miR-155, miR-212, miR-362-3p, miR-518e, and miR-885-5p.

**Fig 5:**
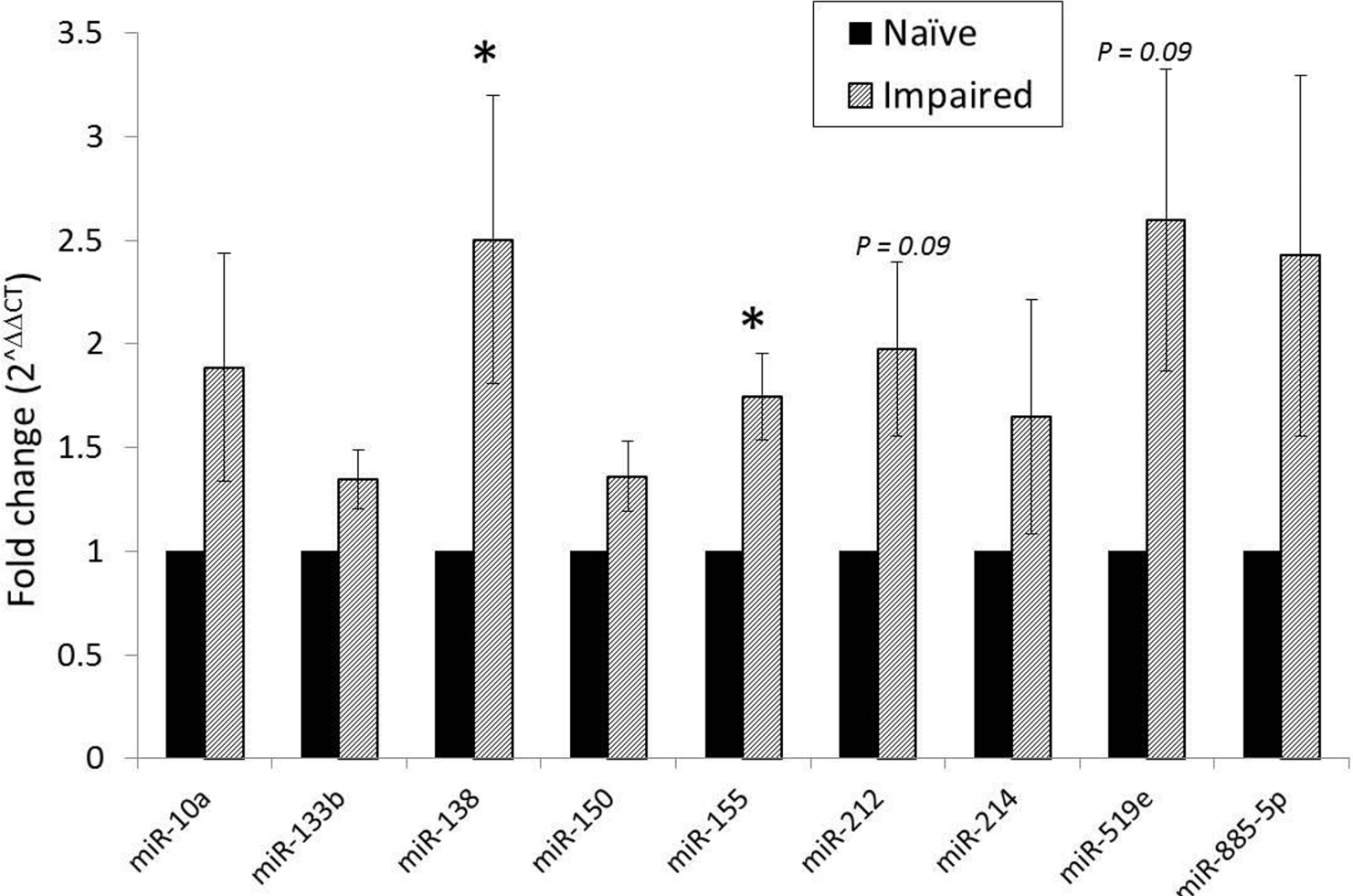
Verification of miRNA differences in the impaired monocyte relative to naïve conditions at 17 h. The most significant miRNAs selected from screening were used for single assay qRT-PCR confirmation. Data were normalized to RNU6B. N = 8. Mean ± S.E.M. *P < 0.05, paired T-test.

To address the risk of false-positives from miRNA screening, we undertook single assay qRT-PCR miRNA confirmation. miRNAs selected for single assay confirmation were chosen based upon fold changes and statistical significance from screening data, as well as biological significance. miR-138 and miR-155 remained significantly upregulated when comparing impaired against naïve conditions at 17 h (Fig 5). miR-212 and miR-519e were also upregulated in the impaired monocyte but did not reach statistical significance (P = 0.09).

### IκK-16 decreased miR-155 expression

**Fig 6:**
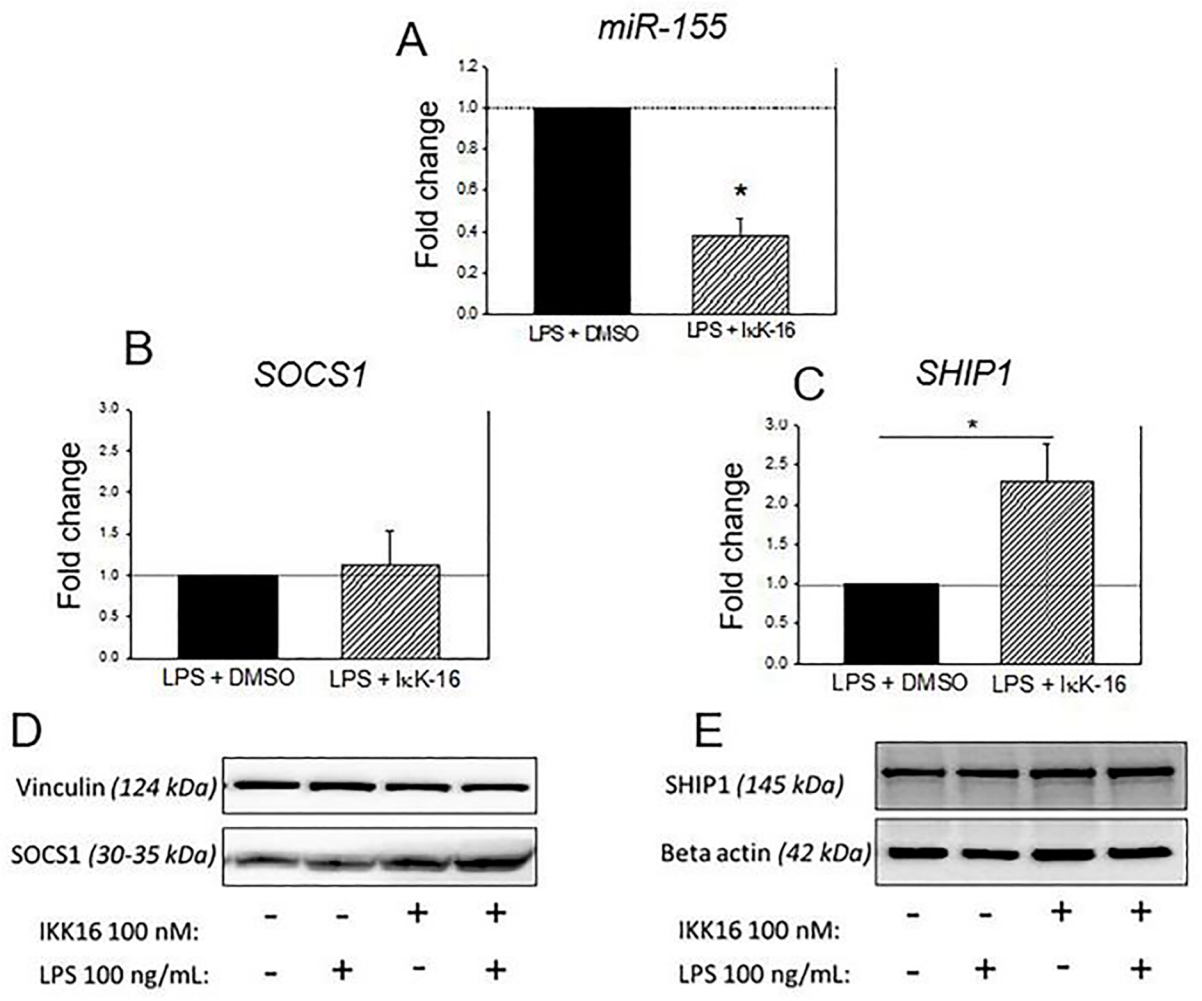
Effect of IκK-16 on miR-155 (A), SOCS1 (B) and SHP1 (C) expression. Monocytes were cultured with 100 nM of IκK-16 or DMSO (control) for 1 h and then 100 ng/mL of LPS was given for 17 h. Levels of miR-155, SOCS1, and SHP1 expression in IκK-16 treated monocytes are expressed as fold change relative to the DMSO (control) monocytes, as determined by qRT-PCR. MiRNA data were normalized to RNU6B, and mRNA data were normalized to 18S. Mean ± S.E.M. N = 6. * P<0.05, paired T-test. Western blot was similarly used determine protein levels at 17 h for SOCS1 (D) and SHP1 (E), with vinculin and beta-actin used as loading controls, respectively.

As a result of IκK inhibition by use of IκK-16 in the presence of 100 ng/mL of LPS, the expression of miR-155 was reduced compared to cells stimulated in the presence of DMSO only (P < 0.05) (Fig 6A). Although no differences in SOCS1 expression were observed (Fig 6B), reciprocal upregulation of SHP1 occurred in monocytes stimulated in the presence of IκK-16 (P < 0.05) (Fig 6C). There was no significant effect of IκK-16 treatment on SOCS1 or SHP1 protein expression at 17 h (Fig 6D and 6E, respectively).

### IκK-16 attenuated the monocyte inflammatory response

**Fig 7:**
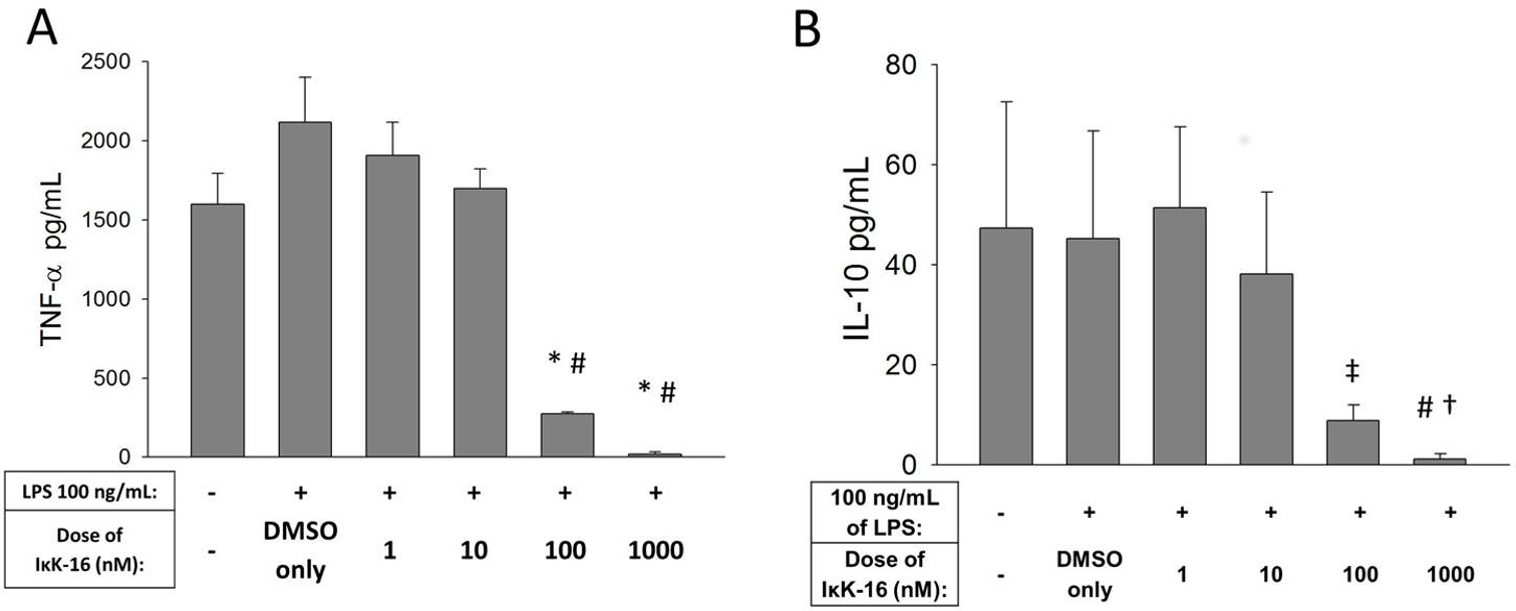
Effect of IκK-16 on cytokine production. Monocytes were treated with DMSO (control) or IκK-16 for 1 h and then stimulated with 100 ng/mL of LPS for an additional 16 h. Supernatant levels of (A) TNF-α and (B) IL-10 were measured by ELISA at 17 h. Mean ± S.E.M. are shown. N = 4. *P < 0.05 vs. DMSO only. †P = 0.06 vs. DMSO only. #P<0.05 vs. 1 nM. ‡P = 0.08 vs. 1 nM. Repeated Measures ANOVA with Tukey test for post-hoc analysis.

In monocytes stimulated with 100 ng/mL of LPS, IκK-16 inhibited the production of both TNF-α and IL-10 in a dose-dependent fashion (Fig 7A & 7B). A dose of 100 nM was sufficient to significantly decrease both cytokines (P < 0.05).

### Effect of IκK-16 on the cytokine response is partially miR-155 dependent

**Fig 8:**
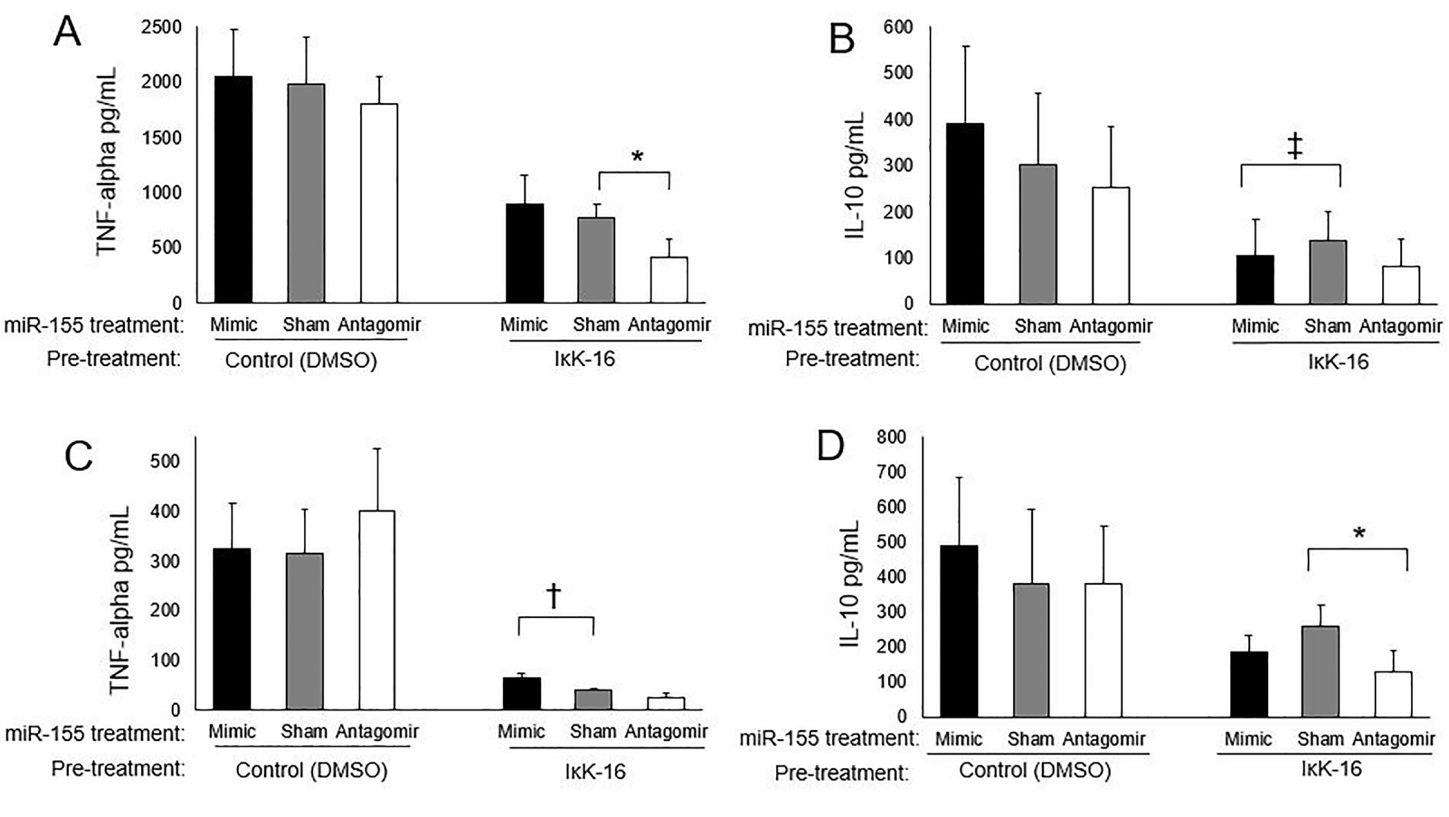
Effect of miR-155 on cytokine production. Monocytes were treated either with DMSO (as control) or 100 nM IκK-16 for 1 h and then transfected with scrambled RNA (sham) or miR-155 mimics or antagomirs for 16 h. Cells were then stimulated with 100 ng/mL of LPS for 6 (A & B) and 24 h (C & D). Supernatant protein levels of TNF-α and IL-10 were determined by ELISA. N = 4, Paired t-test. * P < 0.05. † p = 0.050. ‡ p = 0.090.

To determine whether the inhibitory effect of IκK-16 was miR-155 dependent, gain and loss of miR-155 function experiments were undertaken in the context of IκK-16 treatment. In IκK-16 treated monocytes, transfection of miR-155 mimics increased TNF-α production which reached significance at 24 h after LPS stimulation (P < 0.05, Fig 8C). In the setting of IκK inhibition, transfection with miR-155 antagomirs lead to decreased TNF-α production at 6 h (Fig 8A) and decreased IL-10 production at 24 h (Fig 8D) (P < 0.05).

## Discussion

The immunological response to trauma, major surgery, and sepsis is complex, incompletely understood, and involves multiple possibly redundant mechanisms of regulation. Recent high-throughput studies such as those from the Inflammation and the Host Response to Injury project [3] have given a deeper insight into the “genomic storm” that occurs after major trauma. Rather than a specific marker or cytokine that could be targeted by a “magic bullet,” the findings of these studies have shown that it is the magnitude of the immunological response that dictates the patient outcome. For example, the excessive or prolonged pro-inflammatory response is one aspect of this response, which increases the risk of infection or death in such patients [4, 21]. Epigenetic fine-tuning of such dysregulation is becoming increasingly recognized as an important part of the pathophysiology in which miRNAs play a key role [13, 17]. miR-155 is an example of a post-transcriptional regulatory mechanism that promotes and potentiates such an excessive pro-inflammatory response [15]. In studying new therapies for immunomodulation, we must better understand the molecular mechanisms that underpin its action in order to increase the successful translation to the patient.

In this study, we used a model of impaired monocyte function that characterizes the initial pro-inflammatory response and downregulation of HLA-DR expression, but also typifies the cellular hypo-responsiveness that occurs as a result of excessive stimulation [18, 19]. In our model, suppressed TNF-α gene expression was observed in impaired cells at 2 h following an LPS challenge, with decreased TNF-α protein production by 12 h after the LPS challenge. IL-10 production was also decreased. Indeed, differences in these NFκB-dependent cytokines were demonstrated in the context of altered NFκB signaling in impaired conditions. We believe that these findings are broadly in keeping with previous endotoxin tolerance literature. Our data demonstrated suppressed TRAF-6 and p65 (NFκB) expression in keeping with a suppressed cytokine response following subsequent LPS exposure. miRNA confirmation demonstrated that miR-155 and miR-138 were significantly elevated in the impaired monocyte. Due to the important regulatory role as well as the clinical relevance, we focused on miR-155. The data demonstrated that the upregulation of miR-155 in response to LPS was dependent on the IκK-NFκB pathway. Modulation of the response to a stimulus with IκK-16, a novel specific inhibitor of IκK, was not only capable of modulating miR-155 expression, but also influenced the gene expression of the downstream target of miR-155, SHP1, in a reciprocal fashion. In order to determine whether the inhibitory effect of IκK-16 was miR-155-dependent or not, we undertook gain- and loss-of-function experiments in the setting of IκK-16 inhibition. Indeed, the re-introduction of miR-155 lead to an increase in TNF-α but not in IL-10 production. Conversely, a further decrease in miR-155 via antagomirs decreased both TNF-α and IL-10 levels. These findings suggest that IκK-16 treatment can modulate the excessive pro-inflammatory response, such as that observed in the “high risk” patient, through manipulation of miR-155. Replenishing pools of miR-155 did not completely restore TNF-α levels to that of the non-IκK-16 treated cells. This may indicate that IκK-16 acts through miR-155-independent mechanisms, but may also reflect the dominant effect of IκK inhibition. Another important miRNA, miR-146a, has frequently been found to be associated with endotoxin tolerance [22, 23]. In our screening results, miR-146a appeared to be elevated but did not reach statistical significance. However, we found that a key target of miRNA-146a in toll-like receptor signaling, TRAF-6, was significantly suppressed.

Our results are consistent with previous reports demonstrating an upregulation of miR-155 and miR-138 in response to stimuli such as LPS [24–26]. miR-138 is known to target SIRT1 and HIF-1α, which are two genes that have previously been associated with impaired monocyte responses [27, 28] and could plausibly contribute to the pathogenesis of this form of immunosuppression [29]. This may be an interesting area for future research. The focus of our group has been on studying the role of miR-155 in the monocyte inflammatory response, which was significantly dysregulated miRNA as per our single assay verification experiments. miR-155 has a very complex role through its numerous important gene targets. Previous reports have shown that p65 binds to the promoter region of the miR-155 gene, and that NFκB inhibition prevents miR-155 upregulation [30],[24], Various reports have shown that miR-155 promotes and potentiates the pro-inflammatory response by suppressing SOCS1 and SHP1, thus increasing the pro-inflammatory cytokine response such as TNF-α [24]. Previous reports from our group, along with others, have shown the reciprocal relationship between miR-155 and SOCS1 and SHP1 [16, 31, 32]. The findings of elevated miR-155 in response to the low dose 10 ng/L of LPS at 17 h correlates with elevated TNF-α levels at this time point. However, miR-155 also targets p65, [33] the active subunit of the NFκB pathway, as well as MyD88 and IκK-ε [6, 34]. In this report, we found a correlation between elevated miR-155 and decreased p65. Through delayed suppression of these pro-inflammatory pathways as well as cross-tolerance from the initial rise in TNF-α, miR-155 may also contribute to the impaired monocyte responses [34–36]. Indeed, elevated miR-155 has been reported in patients diagnosed with sepsis [37]. miR-155 may also play a role as a biomarker in patients who have both an excessive pro-inflammatory response and subsequent monocyte impairment that occurs as a result.

It should be noted that inhibition of miR-155 improves outcomes in experimental peritonitis [38, 39]. miRNAs have not yet been successfully used directly as a therapeutic modality in humans; however, some miRNAs are under investigation [40]. What is more feasible is to understand the molecular mechanisms of existing and future therapies by studying their effects on miRNA expression.

Excessive activation of the NFκB pathway in monocytes from surgical patients is associated with an increased risk of postoperative systemic inflammatory response syndrome [41]. There are literally thousands of genes that are up-and downregulated in association with excessive stimulation [42]. However, previous attempts to therapeutically target specific markers or products of excessive inflammation, such as IL-1β and TNF-α, have failed to improve mortality rates [7]. A more feasible approach could be to focus upstream of the cytokines themselves and target pathways such as NFκB that govern immune responses to “tune down” this so-called cytokine storm. IκK-16 is a selective blocker of inhibitor of kappa-B kinase (IκK). Its application in a mouse model of peritonitis attenuated NFκB activation and resulted in decreased multiple organ dysfunction [9]. The same group treated a rat model of hemorrhagic shock with IκK-16, which resulted in decreased circulating cytokines and improved markers of organ function. Most recently, IκK-16 treatment was given to mice with unilateral nephrectomy and peritonitis as a model of chronic kidney disease and sepsis [12]. Decreased cardiac dysfunction and lung inflammation was observed. However, by inhibiting the inflammatory response, one must understand the underlying mechanisms to avoid harmful suppression of the immune response. Promisingly, a murine study of acute lung injury demonstrated that transient IκK inhibition did not compromise bacterial host defense mechanisms [43].

Our results demonstrate that IκK-16 decreases the cytokine levels in primary human monocytes, supporting the findings from these pre-clinical models. In addition, we have shown that IκK-16 decreases miR-155, an important regulator of the immune response, which appears to be one mechanism through which limiting excessive inflammation may be achieved.

This study does have limitations. The process of monocyte isolation is mildly stimulatory to the cells; therefore, the additional pre-treatment of 10 ng/mL of LPS provided only modest fold changes in miRNA expression between conditions. Due to the dynamic nature of intracellular miRNA and mRNA expression during the inflammatory response, we cannot exclude other miRNAs that may be dysregulated at different time points. There are various NFκB inhibitors that have been previously tested in animal models of sepsis. IκK-16 targets IκK-α/β which is proximal in the NFκB pathway. Given the complex cross-talk between different signaling pathways, there may be some benefit in dampening the amplitude of the signal earlier in the pathway compared with agents specifically targeting NFκB. The potential benefits of IκK-16 include that it is not toxic to human cells and has shown benefit in in-vivo sepsis models. Other inhibitors of IκK may also prove to be effective in limiting the magnitude of the immune response.

In summary, we have shown that impaired monocytes express elevated levels of miR-155 and miR-138 and are associated with decreased p65 and TNF-α production. Our data demonstrate that miR-155 expression is IκK-dependent, and that IκK-16 is a novel treatment that appears to modulate the human monocyte inflammatory response through attenuation of miR-155. Understanding the mechanistic effects of IκK-16 on the monocyte inflammatory response may help in translating this potential therapy to the bedside of the acutely unwell patient with an excessive inflammatory response.

## Supporting information

S1 Fig Cell viability is equivalent between naïve and impaired conditions. Cell viability was determined by standard microscopy with Trypan Blue staining. Rates of monocyte viability were equivalent between naïve and impaired conditions. N = 7. N.S., not significant, paired T-test.
(TIF)

S2 Fig Effect of IκK-16 on monocyte viability. Cultured primary monocytes were incubated with increasing doses of IκK16 in the presence of 100 ng/mL of LPS for 16 h. Cells were stained with Trypan Blue and manually counted to determine viability (n = 4).
(TIF)

S1 Table Supplemental individual donor data. (XLSX)
