# Supplementary figures and images for: IκK-16 decreases miRNA-155 expression and attenuates the human monocyte inflammatory response
Source: PLoS One. 2017 Sep 14;12(9):e0183987. doi: 10.1371/journal.pone.0183987 (PMC5598939; doi:10.1371/journal.pone.0183987)

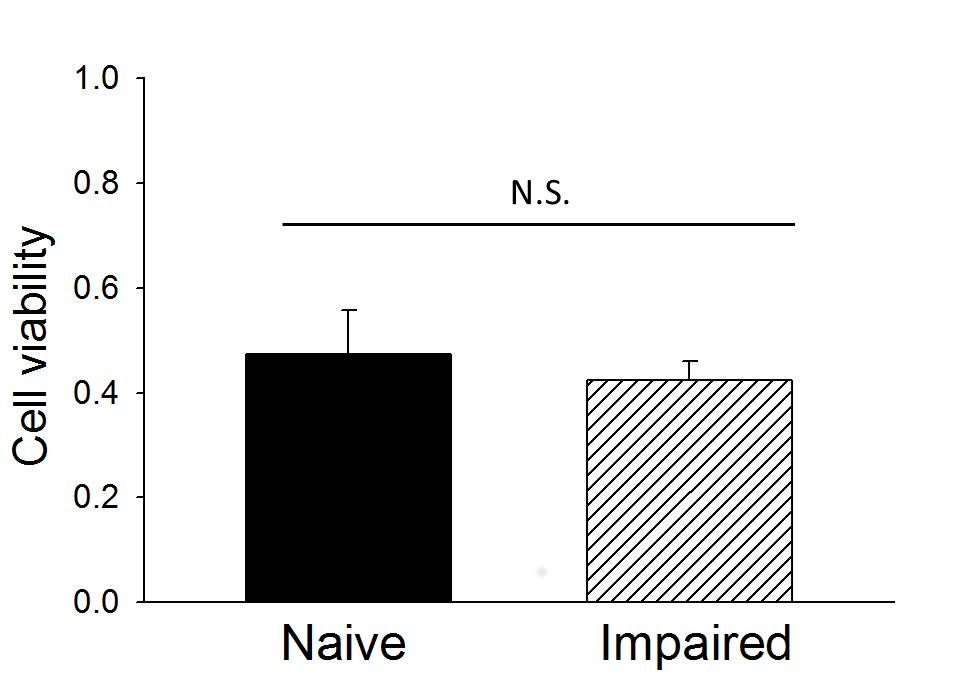

Supplement: S1 Fig — Cell viability was determined by standard microscopy with Trypan Blue staining. Rates of monocyte viability were equivalent between naïve and impaired conditions. N = 7. N.S., not significant, paired T-test. (TIF) [file pone.0183987.s001.TIF]

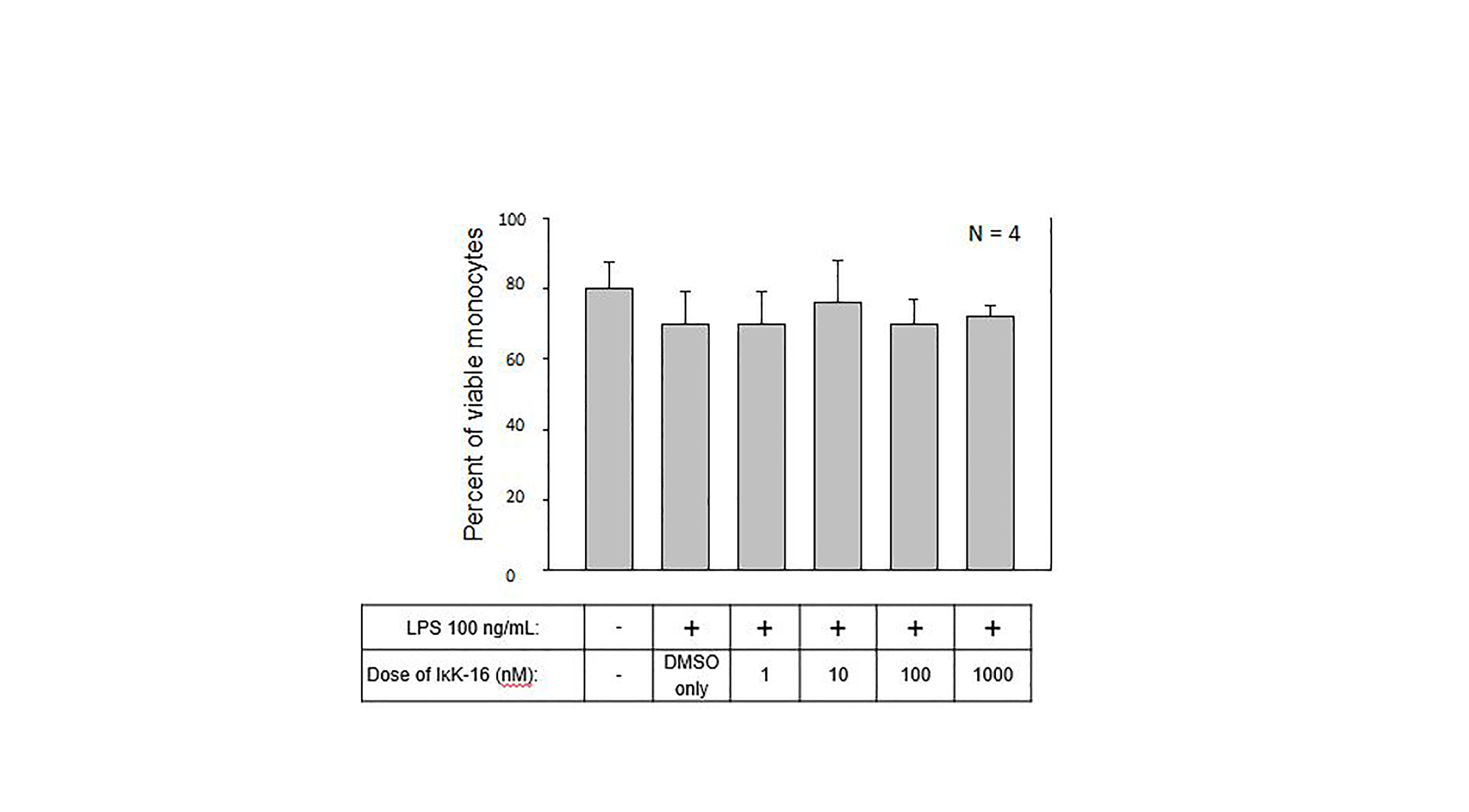

Supplement: S2 Fig — Cultured primary monocytes were incubated with increasing doses of IκK16 in the presence of 100 ng/mL of LPS for 16 h. Cells were stained with Trypan Blue and manually counted to determine viability (n = 4). (TIF) [file pone.0183987.s002.tif]
